# Bacterial versus human thymidylate synthase: Kinetics and functionality

**DOI:** 10.1371/journal.pone.0196506

**Published:** 2018-05-01

**Authors:** Zahidul Islam, Ilya Gurevic, Timothy S. Strutzenberg, Ananda K. Ghosh, Tasnia Iqbal, Amnon Kohen

**Affiliations:** Department of Chemistry, The University of Iowa, Iowa City, IA, United States of America; Monash University, AUSTRALIA

## Abstract

Thymidylate Synthase (TSase) is a highly conserved enzyme that catalyzes the production of the DNA building block thymidylate. Structurally, functionally and mechanistically, bacterial and mammalian TSases share remarkable similarities. Because of this closeness, bacterial enzymes have long been used as model systems for human TSase. Furthermore, while TSase inhibitors have long served as chemotherapeutic drugs, no TSase inhibitor serves as an antibiotic. Despite their high resemblance, the mammalian TSases are distinct in a few known aspects, such as having a N-terminal tail and two insertions in the primary sequence and active/inactive conformations. Here, we aim to comprehensively characterize human (*hs*) TSase and delineate its contrasts and the similarities to the well-studied *Escherichia coli* (*ec*) TSase. We found that, in contrast to *ec*TSase, Mg^2+^ does not enhance reaction rates for *hs*TSase. The temperature dependence of intrinsic kinetic isotope effects (KIEs), on the other hand, suggests that Mg^2+^ has little or no impact on the transition state of hydride transfer in either enzyme, and that the transition state for the hydride transfer in *hs*TSase is looser than in *ec*TSase. Additionally, the substrates’ binding order is strictly ordered for *ec*TSase but slightly less ordered for *hs*TSase. The observed kinetic and functional differences between bacterial and human enzymes may aid in the development of antibiotic drugs with reduced toxicity.

## Introduction

The last committed step in the de novo synthesis of thymidylate (2'-deoxythymidine-5'-monophosphate, dTMP) in most living organisms is catalyzed by the enzyme thymidylate synthase (TSase). Suppression of TSase activity in cells causes a deficiency in the intracellular concentration of thymidylate, bringing DNA reproduction to a halt and thus causing death to cells [1]. The participation of TSase in the biosynthesis of DNA renders this enzyme a suitable target for anticancer drugs [2, 3]. Two well-known drugs, 5-fluorouracil and raltitrexed, that target TSase are prescribed for the treatment of a wide variety of cancers including colorectal, breast and pancreatic tumors [2, 3]. Antibacterial drugs targeting TSase, however, are not in use, in part due to the known structural and functional similarities between the bacterial and human enzymes.

**Fig 1:**
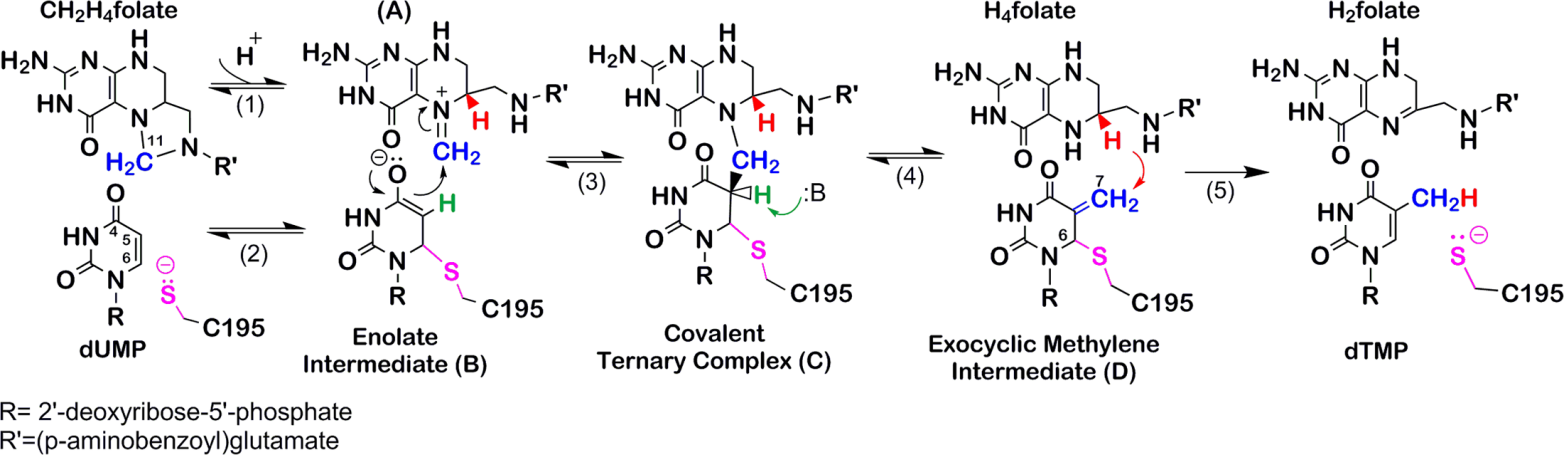
Minimal chemical mechanism of the TSase-catalyzed reaction. Adapted from ref [21].

In the production of dTMP, TSase requires a cofactor, methylene tetrahydrofolate (MTHF), that donates a methylene and a hydride in two separate mechanistic steps; thus, a net methyl group substitutes the C5 hydrogen of the substrate 2'-deoxyuridine-5'-monophosphate (dUMP) (Fig 1) [1, 4]. In the mechanism of *ec*TSase, an active-site cysteine makes a nucleophilic attack at the C6 of dUMP, forming a covalent TSase-dUMP enolate (step 2 in Fig 1). The enolate attacks the pre-activated, iminium form of MTHF, leading to the formation of a covalently bonded enzyme-substrate-cofactor ternary complex, TSase-dUMP-MTHF (compound C in Fig 1). Abstraction of the C5 proton by an active site general base causes dissociation of MTHF as tetrahydrofolate (THF) and formation of an exocyclic methylene intermediate (compound D in Fig 1). Finally, a hydride from THF is transferred to the C7 of intermediate D, accounting for the products dTMP and dihydrofolate (DHF).

**Fig 2:**
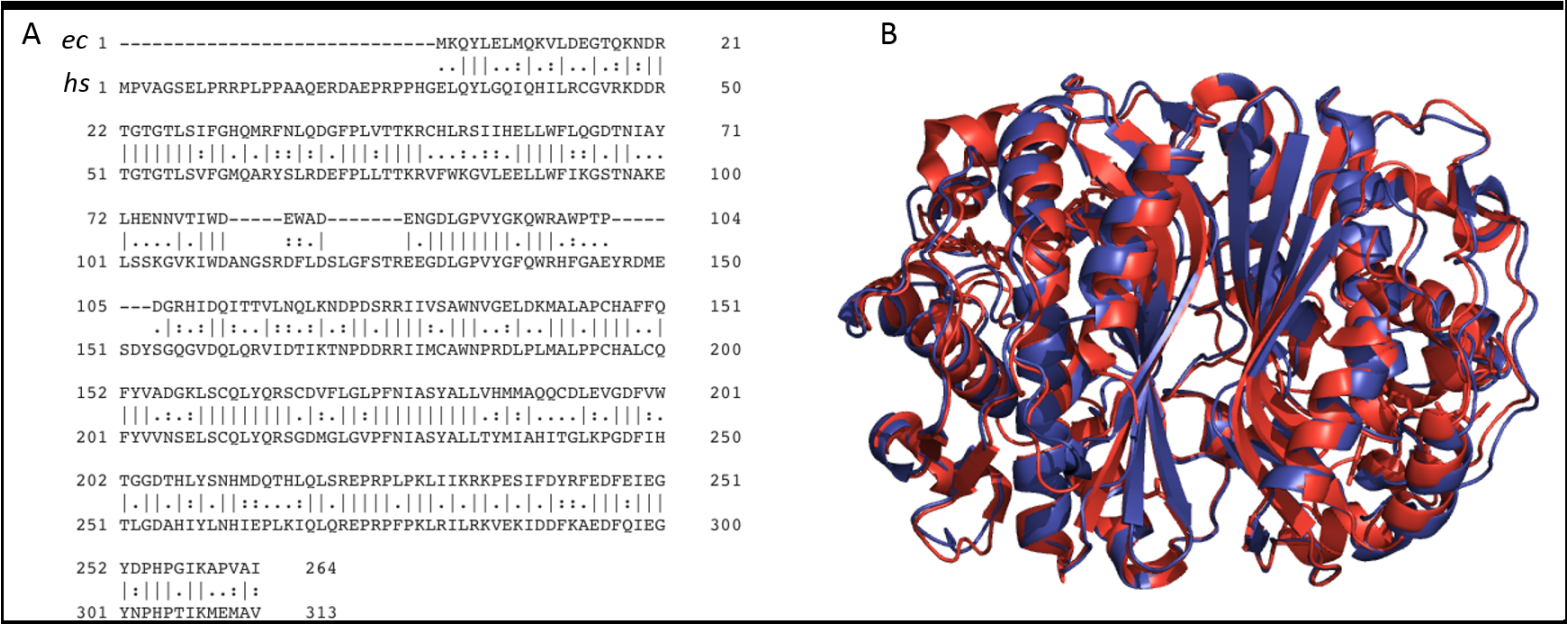
(A) Primary sequence alignment between *ec* and *hs* TSase. Vertical lines, colons and dots represent identity, similarity and mismatch, respectively. (B) Structural alignment between whole *ec*TSase (blue) and *hs*TSase (red, where the N-terminal is not determined). PDB ID 2KCE (*ec*) and 1HVY (*hs*).

TSase is one of the most highly conserved enzymes known–from the very primitive organisms to humans. *E*. *coli* (*ec*) and human (*hs*) TSase share 46% and 60% sequence identity and similarity, respectively (Fig 2, panel A). Residues in the active site, substrate binding region, and many of the remote residues are also found to be highly conserved across the spectrum of life that encodes the *thyA* gene [5, 6]. Both secondary and tertiary structures of *ec* and *hs* TSase align quite well with each other (Fig 2, panel B). Because of these similarities in sequences, structures and functions, bacterial TSase has long been used as a model system for drug design targeting the human enzyme.

However, despite structural and functional similarities between bacterial and human TSase, the mammalian TSases are distinct in a few aspects. First of all, mammalian TSases have evolved to incorporate multiple insertions into different regions of the polypeptide chain: an extension of the N-terminus by 29 residues and an insertion of 12 and 8 residues at positions 117 and 146, respectively (positions mentioned refer to the *hs*TSase sequence unless otherwise stated). A contrast between bacterial and human TSase is also noted in protein conformations [7]. While *ec*TSase crystallizes only in the closed conformation both in the presence and the absence of ligands, in crystal structures of *hs*TSase the loop comprising residues 181–197, which includes the catalytic nucleophile cysteine 195, is rotated 180° out of the active site in its unliganded state. This is referred to as the inactive conformation. The binding of substrates or their analogues induces a conformational change across the protein that rotates this loop towards the active site, transforming the enzyme into an architecture poised for catalysis. Conformational switching causes other structural changes as well, such as bringing the loop comprising residues 107–128, which includes one of the eukaryotic insertions, from a disordered to an ordered structure as the enzyme goes from its inactive to its active configuration [7]. It has long been known that Mg^2+^ enhances the turnover number of bacterial TSase; however, this effect has not been tested for mammalian TSases [8, 9]. To further explore discrepancies between microbial and *hs*TSase, a thorough kinetic and mechanistic investigation of *hs*TSase is warranted. A more detailed characterization of *hs*TSase might reveal crucial properties, different from bacterial ones, that would aid in the design of non-toxic antibacterial drugs [5]. Exploitation of fine differences between the *E*. *coli* and human enzymes has precedent [5]. Namely, one report featured the development of dansyl hydrazine, dansyl tyrosine and didansyl tyrosine as specific folate-competitive inhibitors of human, *Lactobacillus casei* and *E*. *coli* TSases, respectively, with modest potency (>1 μM) but approximately 1–2 orders of magnitude of selectivity. This suggests that additional discrete properties of *hs*TSase could be utilized for designing more potent, more selective finely-tuned new chemotherapeutic drugs.

TSase exerts a tight control over its intracellular expression during cell cycles. This enzyme binds its own mRNA, repressing its translation. Thereby, the organism achieves a negative autoregulation [10–13]. The *apo* state of TSase promotes the formation of protein-mRNA complex. For positive autoregulation, binding of substrates or substrate analogues disrupts the formation of TSase-mRNA, causing translational derepression and synthesis of more protein. Translational autoregulation of protein synthesis is prevalent in bacteria, but such feedback regulation by binding to mRNA is rare in eukaryotes [10]. In humans, the TSase system represents the first reported instance of such translational autoregulation [10]. Control over expression and function of TSase may also be achieved by posttranslational modifications [14]. In contrast to the bacterial one, mammalian TSases are reportedly known to undergo certain posttranslational modifications in certain cell lines under certain conditions [14]. Those modifications could include methylation, phosphorylation [14] and/or acetylation of the N-terminal methionine [15]. It was reported that *hs*TSase could under particular circumstances have methylated glutamates and phosphorylation at serine. However, neither the modification sites nor the functional effect of those modifications, nor the conditions under which these forms are generated, have been unequivocally documented [14, 16, 17]. While a plethora of kinetic, structural and mechanistic studies are available on bacterial TSase [1, 4, 8, 18–22], mammalian TSase lacks such deep interrogations. Here we aim at a more comprehensive characterization of recombinant *hs*TSase. We report multiple kinetic investigations with and without Mg^2+^, substrate binding order studies, and assessment of intrinsic KIEs and their temperature dependence for the proton and hydride transfer (steps 4 and 5 in Fig 1). The contrasts and similarities in function and kinetics between *ec* and *hs* TSase are presented and discussed. The KIE studies provide a sense of the active site architecture. In general, KIE results lead to information on distance considerations that guide medicinal chemists toward atomic replacement and / or spacers like methylene groups. There is precedent for the design of a femtomolar transition state analogue inhibitor for the enzyme purine nucleoside phosphorylase based in large part on the determination of KIEs.[23, 24] For instance, if the human TSase were found to be larger, the active site could be expanded by atomic replacement, i.e. including a larger atom; for example, N5 of the folate could be replaced with a phosphorus atom in a non-reactive MTHF analog to make the ground state of the resulting ternary complex more like the transition state for hydride transfer. The non-reactive MTHF analog inhibitor for *E*. *coli* TSase could replace N5 with an atom of similar size (for example, carbon).

## Materials and methods

### Materials and instruments

Ni-NTA Superflow resin was purchased from Qiagen. GE Healthcare Life Sciences was the source of the PD-10 desalting columns filled with Sephadex G-25 resin. LB powder was purchased from Research Products International, Inc. [5-^3^H]-dUMP, specific radioactivity ~14 Ci/mmol (for proton abstraction KIEs) and [2-^14^C]-dUMP, specific radioactivity ~53 mCi/mmol, for hydride transfer experiments were from Moravek Biochemicals. Unlabeled MTHF was from Merck. Radiolabeled MTHF samples–both H/T and D/T–were synthesized by following published procedures from previous publications [25, 26]. Ultima Gold liquid scintillation (LS) cocktail was from PerkinElmer, and Research Products International was the source of the LS vials. LS counting was performed on a Packard TRI-CARB 2900 TR instrument. Separations of reaction mixtures were conducted on reverse-phase Supelco Discovery C18 columns on Agilent Technologies 1100 HPLC systems. Steady-state kinetics were studied on a Hewlett-Packard Model 8452A diode-array UV-vis spectrophotometer connected to a water bath for temperature control. Analysis of steady-state kinetic data for *hs*TSase was performed using GraphFit software.

### Protein expression and purification

Vector pQE80L containing the TYMS gene was a generous gift from Professor M. Paola Costi and Reggio Emilia, University of Modena, Italy. We sub-cloned the gene into pET28a(+) containing a thrombin cleavable polyhistidine. We inserted the TSase gene between NdeI and BamHI restriction sites of pET28a(+) plasmid. The sub-cloned pET28a(+) encoding His_6_-*hs*TSase and kanamycin resistance as a selection marker was transformed into *E*. *coli* BL21(DE3) cells. Plasmid was extracted from several colonies and sequenced to verify proper transformation, and these colonies were propagated and preserved as 40% glycerol stocks at -80°C. After overnight growth at 37°C of a primary culture of ~50 mL supplemented with kanamycin at a final concentration of 40 mg/L, inoculation into four flasks of 1.5 L bulk culture LB media in each containing kanamycin at a final concentration of 40 mg/L was performed at a 1:150 ratio. After growth to an O.D. at 600 nm of approximately 0.8, IPTG was added to a final concentration of 1 mM, initiating overexpression of the target protein overnight (~12 hrs). After harvesting cells by centrifugation at ~5000 rpm for 30 minutes at 4°C, the pellets were frozen at -80°C until further processing; ~2 g of cells were obtained per liter of bulk culture.

Cell pellets–typically from 3 L of bulk culture–were resuspended in 4 mL of resuspension buffer (25 mM potassium phosphate, 30 mM NaCl, pH = 7.5) per gram of original cell mass with continuous stirring; this and all subsequent steps were performed at 4°C. Once the pellet was resuspended (~30 minutes), the cells were lysed by passing through the French Press apparatus twice. The lysate was centrifuged at 15,000 rpm for 30 min, after which the supernatant was retained and the cell debris discarded. The lysate was subjected to gentle rocking with ~ 1 mL of Ni-NTA Superflow resin per gram of original cell mass for one hour. The mixture was applied to a column pre-packed with ~0.5 mL of Ni-NTA Superflow resin per gram of original cell mass and pre-equilibrated in wash buffer (50 mM potassium phosphate, 25 mM imidazole, pH 7.5). After collecting the flow-through, five column volumes (CV) of wash buffer were passed through the column, collecting 5 mL fractions and testing them by visual Bradford assay for protein content until no noticeable protein remained. Next, 5 CV of elution buffer (50 mM potassium phosphate, 250 mM imidazole, pH 7.5) were passed through the column, and eluent was collected in 1–3 mL fractions, until no protein was evident in the eluent. The eluent was then buffer-exchanged into 25 mM potassium phosphate, 30 mM NaCl, pH 7.5, by using a PD-10 desalting column. The concentration of protein in the resulting solution was measured by Nanodrop using ε_*280*_ = 43,130 M^-1^ cm^-1^. (To prepare the column for reuse, ~2 CV of 1 M imidazole, pH 8, ~4 CV water, ~2 CV 0.5 M NaOH, ~4 CV water and then ~2 CV 70/30 water/ethanol were passed through. Storage was in the 70/30 water/ethanol mixture.)

In order to remove the thrombin-cleavable hexahistidine tag, the solution of *hs*TSase from the previous step and thrombin were combined in a final concentration of 0.75 U thrombin/(mg protein), in thrombin cleavage buffer provided by GE Healthcare. The concentration of *hs*TSase was approximately 5 mg/ml and the mixture was incubated at 4°C with rocking overnight (~12 hrs). A Ni-NTA column was packed and equilibrated in wash buffer (composition as above). The solution was applied to the column; flow-through, containing the target, tag-free *hs*TSase, was collected and saved. Next, ~5 column volumes (CV) of wash buffer were passed through the column until the visual Bradford assay indicated no protein in the wash. The flow-through was concentrated via Amicon 10 kDa cutoff concentrators and buffer-exchanged into 25 mM potassium phosphate pH 7.5; then ethylene glycol was added to 10% of the volume. The final concentration of enzyme was around 300 μM, as determined by Nanodrop using ε_*280*_ = 43,130 M^-1^ cm^-1^ and was stored in small (~50 μl) aliquots at -80°C. The presence of the tag-free *hs*TSase was confirmed by MALDI-TOF mass spectrometry.

### Steady-state parameters for WT *hs*TSase with and without Mg^2+^

Stock solutions of 10 mM dUMP in 100 mM tris, pH 7.5, and 10 mM MTHF in ascorbate-citrate buffer with 10 mM HCHO and 4 mM TCEP were prepared. Standardization of the concentration of the MTHF stock was performed by combining a known volume with excess dUMP in reaction buffer (100 mM tris pH 7.5 buffer, with 1 mM tris(carboxyethyl) phosphine (TCEP) to prevent oxidation of cysteines, 50 mM MgCl_2_, and 7 mM HCHO) in a quartz cuvette of 1 cm pathlength in the HP UV-vis spectrophotometer. Next, the initial A_340_ was noted, and enzyme was added to initiate the reaction followed by thorough mixing. The difference in A_340_ between the initial and plateau levels was converted to concentration present initially using Δε_*340*, *MTHF-DHF*_ = 6,400 M^-1^ cm^-1^ [26]. The enzyme concentration was quantified by diluting the enzyme stock in reaction buffer and using ε_*280*_ = 43,130 M^-1^ cm^-1^ as determined from web.expasy.org/protparam/.

Steady-state kinetics were studied by using reactions in the wells of a 96-well plate in an Epoch plate reader at approximately 22°C. The total reaction volume in each well was 300 μL, with 150 μL of 2x assay buffer (200 mM tris pH 7.5, 2 mM TCEP, 14 mM HCHO), 30 μL of dUMP solution at the appropriate concentration, 30 μL of MTHF solution at the appropriate concentration, 30 μL of ddH_2_O or of 0.5 M MgCl_2,_ depending on the experimental condition, 30 μL of ddH_2_O and initiated with 30 μL of 150 nM *hs*TSase. In order to determine the appropriate pathlength, reactions were initiated with varied [MTHF] and no dUMP. The steady A_340_ was divided by the concentration of MTHF and by the previously reported ε_*340*, *MTHF*_ = 1,010 M^-1^ cm^-1^ [27] to find *b =* 0.87 cm. Each run included three rows of 8 wells, initiated at the same time; each condition was repeated in triplicate. At a final concentration of 100 μM MTHF, dUMP was varied from 1 μM to 100 μM final concentration. At a final concentration of 100 μM dUMP, MTHF was varied from 2 μM to 500 μM. Scans were performed every few seconds over the course of 15 minutes. Linear regions over the first few minutes of the experiment were selected for analysis and fit to a line, whose slope was divided by Δε_*340*, *MTHF-DHF*_ = 6,400 M^-1^ cm^-1^ [26] and the pathlength to find the initial velocity in concentration per second. Goodness of fit statistics were favorable, R^2^ > 0.9, RSD < 10%. These initial velocity values were then divided by enzyme concentration.

For the data analysis, the rates (divided by the total enzyme) for all three replicates were included in performing the fitting. For fixed MTHF and varied dUMP, a simple Michaelis-Menten model was used:

$$\frac{V}{{\left[E\right]}_{t}}=\frac{k_{cat}[dUMP]}{K_{m,dUMP}+[dUMP]}$$ (1)

Here, V refers to the initial velocity, [E]_t_ is the total enzyme concentration, *K*_*m*, *dUMP*_ is the Michaelis constant for dUMP and *k*_cat_ is the enzyme’s turnover number. However, the studies with fixed dUMP and varied MTHF were fitted to a model that accounts for substrate inhibition by MTHF, with the choice of Hill coefficient = 1 [8]:

$$\frac{V}{{\left[E\right]}_{t}}=\frac{k_{cat}[S]}{K_{m,MTHF}+[S](1+\frac{\left[S\right]}{K_{S}})}$$ (2)

Here, V refers to the initial velocity, [E]_t_ is the total enzyme concentration, *K*_*m*,*MTHF*_ is the Michaelis constant for MTHF, *k*_cat_ is the enzyme’s turnover number and *K*_*S*_ is the substrate inhibition constant for MTHF. Although the fit was to all of the replicates, only the means and standard deviations at each condition are shown in the plots along with the fitted curve.

### Proton abstraction H/T kinetic isotope effects for *hs*TSase while varying [MTHF]

Reaction mixtures were prepared in 100 mM tris pH 7.5 buffer, with 1 mM TCEP (antioxidant), 50 mM MgCl_2_, and 7 mM HCHO. A mixture of [5-^3^H]-dUMP and [2-^14^C]-dUMP was prepared such that the ratio of ^3^H:^14^C was between 5 and 9, as the lower average energy of β particles from ^3^H than ^14^C necessitates higher tritium radioactivity for accuracy of LSC counting. The total [dUMP] was approximately 3 μM, while [MTHF]–present in excess in all cases–was varied, ranging from the low μM to the mM range. The pH of each mixture was adjusted at 25°C. There was ~ 500 μL of reaction mixture total, with approximately 150 kilodisintegrations per minute (kdpm) ^3^H and 20 kdpm ^14^C per ~ 30–40 μL aliquot. A sample of ~ 30–40 μL representing t_zero_, in order to identify the tritium originally in water, was taken for each reaction. Then enzyme, diluted in 100 mM tris at pH = 7.5, was added to ~10% of the final volume. The final enzyme concentrations were typically in the 10–50 nM range. Aliquots were mixed with ~ 10 μL of 5-fluoro dUMP inhibitor solution (at least 10-fold molar excess over dUMP) and frozen in liquid nitrogen at times that correspond to 20–80% fractional conversion (*f*) as assessed by ^14^C radioactivity ($100\%∙\frac{{}^{14}C-dTMP}{{}^{14}C-dUMP{+}^{14}C-dTMP}$) upon HPLC separation. Approximately ten samples were taken over the course of the reaction (time points, usually within an hour of initiating the reaction); after the last one, a large concentration of WT *hs*TSase was added, and the mixture was incubated an additional 30–45 min to ensure no starting materials remained (infinity points). Ratios of tritium in water–corrected for initial tritium in water–to ^14^C in dTMP, $\frac{3_{H\;in\;water}}{14_{C\;in\;dTMP}}$, at time t (R_t_) and infinity (R_inf_) were computed, with at least four time points and two infinities per concentration of MTHF. The relevant values of ${KIE}_{obs}=\frac{ln(1-f)}{ln(1-f\frac{R_{t}}{R_{inf}})}$ were extracted [28]. The observed KIEs in this one-pot, competitive measurement are the ratio of the catalytic efficiencies ($\frac{k_{cat}}{K_{m}}$) for the light isotopologue of the substrate over the heavy isotopologue of the substrate.

### Proton abstraction D/T and intrinsic kinetic isotope effects for *hs*TSase

In general, procedures here closely followed those previously published [22]. The competitive D/T KIE experiment was performed at the same concentrations of substrates (~ 3 μM total [dUMP] and ~ 4 μM [MTHF]), similar concentrations of enzyme and in the same manner as the H/T experiment. The labeled nucleotides for the D/T experiment–[5-^3^H]-dUMP and [5-^2^H, 2-^14^C]-dUMP–were prepared as reported previously [29, 30]. The analysis and interpretation of our data tracks with published reviews [31, 32]. All possible combinations of observed KIEs were employed to obtain intrinsic KIEs according to the Northrop equation [33]

$$\frac{{1/KIE}_{obs}^{HT}-1}{1/{KIE}_{obs}^{DT}-1}=\frac{1/{KIE}_{int}^{HT}-1}{{{1/(KIE}_{int}^{HT})}^{0.3}-1}$$ (3)

This equation, in which ${KIE}_{int}^{HT}$ is the only unknown, has no analytical solution but can be solved via numerical methods (an online version is found at https://chem.uiowa.edu/kohen-research-group/calculation-intrinsic-isotope-effects) [28, 33]. A plot of ${KIE}_{int}^{HT}$ versus *1/T* was generated and fitted, using Kaleidagraph 4.5.3 software, to the Arrhenius equation:

$${KIE}_{int}=\frac{k_{H}}{k_{T}}=\frac{A_{H}}{A_{T}}exp\left(-\frac{{\Delta E}_{a(T-H)}}{RT}\right)$$ (4)

where ΔE_a (T-H)_ and A_H_/A_T_ represent isotope effects on the activation energy and on the pre-exponential factor, respectively, and H and T denote hydrogen and tritium, respectively. All the intrinsic KIE values were employed to obtain the fitting parameters, but only average intrinsic KIEs at each temperature are depicted in the graph.

### Hydride transfer kinetic isotope effects

In general, procedures here followed closely along the lines of those previously published [8]. Reaction mixtures were again in 100 mM tris pH 7.5 buffer, with 1 mM TCEP to prevent oxidation of cysteines, 50 mM MgCl_2_, and 7 mM HCHO. In buffer without Mg^2+^, a final concentration of 1 mM EDTA was used to prevent adventitious Mg^2+^ from exerting a confounding effect on our results. For H vs. T competitive experiments, mixtures of (*R*)-6-[^3^H]-MTHF + (*R*)-6-[^1^H]-MTHF + 2-[^14^C]-dUMP were prepared, while for D vs. T competitive experiments, mixtures of (*R*)-6-[^3^H]-MTHF + (*R*)-6-[^2^H]-MTHF + 2-[^14^C]-dUMP were prepared, such that the ratio of ^3^H:^14^C was between 4 and 9. The total [MTHF] was ~ 80–120 μM, while total [dUMP]–present in excess in all cases–was ~ 100–150 μM. (This excess was verified by incubating a small amount of reaction mixture with WT TSase and HPLC separation and LSC analysis). The pH of each mixture was adjusted at the appropriate temperature (5°C, 15°C, 25°C, 35°C). There was ~ 500 μL of reaction mixture total per temperature, with approximately 150 kdpm ^3^H and 20 kdpm ^14^C per aliquot. An aliquot, typically ~ 30–40 μL, was taken to assess the initial state of the reaction mixture, referred to as t_zero_. Then enzyme, diluted in 100 mM tris, pH = 7.5, was added to ~10% of the final volume. The final enzyme concentrations were typically in the 50 nM to 1 μM range. Aliquots were mixed with ~ 10 μL of 5-fluoro dUMP inhibitor solution (at least 10-fold molar excess over dUMP) and frozen in liquid nitrogen at times that corresponded to 20–80% normalized fractional conversion (*f*_*nml*_) as assessed by ^14^C radioactivity ($f_{raw}=100\%∙\frac{{}^{14}C-dTMP}{{}^{14}C-dUMP{+}^{14}C-dTMP}$) upon HPLC separation, with $f_{nml}=100\%∙\frac{f_{raw}}{f_{inf}}$. Approximately ten samples were taken over the course of the reaction (time points); after the last one, a large concentration of WT *hs*TSase was added, and the mixture was incubated an additional 30–45 min at 25°C to ensure that the reaction went to completion (infinity points). Ratios of tritium in dTMP to ^14^C in dTMP, $\frac{3_{H\;in\;dTMP}}{14_{C\;in\;dTMP}}$, at time t (R_t_) and infinity (R_inf_) were computed, with at least four time points and three infinities per temperature. The relevant values of ${KIE}_{obs}=\frac{ln(1-f_{nml})}{ln(1-f_{nml}\frac{R_{t}}{R_{inf}})}$ were extracted [28]. Reviews of the analysis and interpretation methods for these data sets have been previously published [31, 32]. At each temperature, all possible combinations of *KIE*_*obs*_ were substituted into Northrop Eq (3) above to obtain a set of ${KIE}_{int}^{HT}$ by numerical methods. A plot of ${KIE}_{int}^{HT}$ versus *1/T* was generated and fitted, using Kaleidagraph 4.5.3 software, to the Arrhenius Eq (4), obtaining ΔE_a (T-H)_ and A_H_/A_T_, as described above for proton abstraction. All the intrinsic KIE values were employed to obtain the fitting parameters, but only the average intrinsic KIEs at each temperature are depicted in the graph.

## Results and discussion

### Effect of Mg^2+^

#### Mg^2+^ enhances rates of *ec*TSase but not *hs*TSase

Previous studies reported that Mg^2+^ accelerates the rate of *ec*TSase by seven-fold and increases the Michaelis constant (K_M_) for dUMP by ∼5-fold at 25°C–although K_m_ reflects more steps than just binding [8, 9]. MTHF substrate-inhibition is known for *ec*TSase because of an alternative unproductive binding mode of the cofactor [18, 34]. The presence of Mg^2+^ affected the cooperativity in MTHF binding, suggesting Mg^2+^ mediates the interaction between MTHF and the *ec*TSase [8]. To examine the effect of Mg^2+^ on *hs*TSase the same steady state parameters were measured in the absence and presence of 50 mM Mg^2+^–the same concentration used in the studies for *ec*TSase. In contrast to bacterial TSase, the steady-state experiments showed no effect of Mg^2+^ on *hs*TSase (Table 1). Michaelis-Menten plots appear in S1 and S2 Figs. The *hs*TSase measurements compare favorably with ref [35]. The catalytic efficiency of mammalian TSase with respect to dUMP was found to be about 10-fold lower than that for bacterial TSase irrespective of Mg^2+^. Like *ec*TSase, *hs*TSase exhibits MTHF substrate-inhibition, but in contrast to *ec*TSase, this was not affected by Mg^2+^. Thus, it seems that in contrast to *ec*TSase the presence of Mg^2+^ had little or no influence on steady state parameters for *hs*TSase. In *ec*TSase, Mg^2+^ was found to bind between the glutamate tail of MTHF and the surface of the protein, appearing to thereby mediate a hydrogen bond network that extends from its binding region (residues 76 to 93 in *ec*TSase) to the active site of the protein [8]. This communication of Mg^2+^ through the H-bond network to the catalytic site might contribute to the rate enhancements observed for *ec*TSase. However, the residues that constitute the Mg^2+^ binding region in *ec* are not conserved in *hs*TSase; therefore, Mg^2+^ may not have an opportunity to bind the protein at the corresponding site of *hs*TSase.

**Table 1:** Steady-state kinetic parameters of *ec* and *hs* TSase in the presence and absence of Mg^2+^ (S1 and S2 Figs).

Parameter	Enzyme	w Mg^2+^	w/o Mg^2+^
*k*_*cat*_ (s^-1^)	*ec*^a^	8.7 ± 0.2	1.32 ± 0.02
	*hs*	0.54 ± 0.03	0.52 ± 0.02
K_*m*_^*MTHF*^ (μM)	*ec*^a^	15 ± 1	17 ± 2
	*hs*	4.5 ± 0.8	2.9 ± 0.4
K_*m*_^*dUMP*^ (μM)	*ec*^a^	2.4 ± 0.2	0.5 ± 0.1
	*hs*	2.5 ± 0.8	4.2 ± 0.8
*k*_*cat*_*/K*_*m*, *dUMP*_ (s^-1^μM^-1^)	*ec*^a^	3.6 ± 0.3	2.6 ± 0.6
	*hs*	0.22 ± 0.07	0.12 ± 0.02
*k*_*cat*_*/K*_*m*, *MTHF*_ (s^-1^μM^-1^)	*ec*^a^	0.58 ± 0.04	0.08 ± 0.01
	*hs*	0.12 ± 0.02	0.18 ± 0.03

^a^ref [8, 18].

#### Mg^2+^ has no impact on the transition state structure of hydride transfer

Previous studies reported that in the absence of Mg^2+^ the hydride transfer in *ec*TSase is rate-limiting on both the first order (*k*_*cat*_) and the second order rate constants (*k*_*cat*_/K_*m*_) [18, 36]. Since Mg^2+^ accelerates the turnover rate and makes the hydride transfer non-rate-limiting, it seems that Mg^2+^ contributes to the activation of hydride transfer in *ec*TSase. For *hs*TSase, on the other hand, Mg^2+^ does not have any impact on the turnover number. However, to check if Mg^2+^ has any impact on the organization of the active site for the hydride transfer, we examined the intrinsic kinetic isotope effect (KIE) on this step with and without Mg^2+^ by following the same competitive method used for *ec*TSase. KIE is a useful probe for assessing a specific kinetic step in the cascade of physical and chemical events [32, 37–39]. We found that Mg^2+^ has a marginal effect on the observed KIEs in *hs* TSase, but no impact on its intrinsic value (Table 2). The lack of change in intrinsic KIEs indicates that Mg^2+^ has no influence on the transition state structure of hydride transfer in both bacterial and human TSases. Taken together, although *ec* and *hs* TSase are structurally and mechanistically very much alike, the function and kinetic parameters of only *ec*TSase seem to be affected by Mg^2+^.

**Table 2:** Observed and intrinsic KIEs of *ec* and *hs* TSase in presence and absence of Mg^2+^.

Parameter	Enzyme	w Mg^2+^	w/o Mg^2+^
KIE_obs_ (H/T)	*ec*^a^	4.14 ± 0.02	7.07 ± 0.03
	*hs*	3.05 ± 0.03	3.19 ± 0.03
KIE_obs_ (D/T)	*ec*^a^	1.65 ± 0.01	1.81 ± 0.04
	*hs*	1.61 ± 0.02	1.64 ± 0.01
Intrinsic KIE (H/T)	*ec*^a^	7.4 ± 0.9	7.4 ± 0.5
	*hs*	11.0 ± 0.9	11.6 ± 0.8

^a^from ref [8, 18].

#### Substrate binding order in *E*. *coli* vs. human TSase

To convert dUMP to dTMP, TSase requires MTHF that provides a net methyl. Binding of the dUMP to *ec*TSase prompts binding of MTHF to the protein, leading to the formation of the ternary complex [40]. This sequence is likely facilitated by the fact that *ec*TSase maintains a very strict substrate-binding order: dUMP is the first to bind, followed by MTHF [19].

To test whether this is also true for *hs*TSase we measured the effect of MTHF concentration on the observed KIEs measured with isotopically labeled dUMP as reported for the *ec*TSase [19]. In the chemical conversion of dUMP to dTMP, a proton abstraction from C5 of dUMP (step 4 in Fig 1) takes place, and KIEs on this proton abstraction are a sensitive tool to investigate the order of substrate binding [19]. In these measurements, the substrate dUMP is labeled with either tritium at its C5 or ^14^C at its C2 (see Materials and Methods). For H/T observed KIEs (i.e., *k*_cat_/*K*_M_ for C5-protiated dUMP divided by *k*_cat_/*K*_M_ for C5-tritiated dUMP), a mixture of [5-^3^H]-dUMP, [2-^14^C, 5-^1^H]-dUMP and unlabeled MTHF is used. In a strictly ordered binding mechanism as in *ec*TSase, where dUMP is the first to bind, a high concentration of the second-binding ligand, MTHF, obstructs the isotopic differentiation by the protein because of the commitment of the ternary complex to go forward [19, 29]. Thus, in an ordered binding mechanism, a high MTHF concentration causes the observed KIE for proton abstraction to approach unity as described in detail in refs [19] and [22].

**Fig 3:**
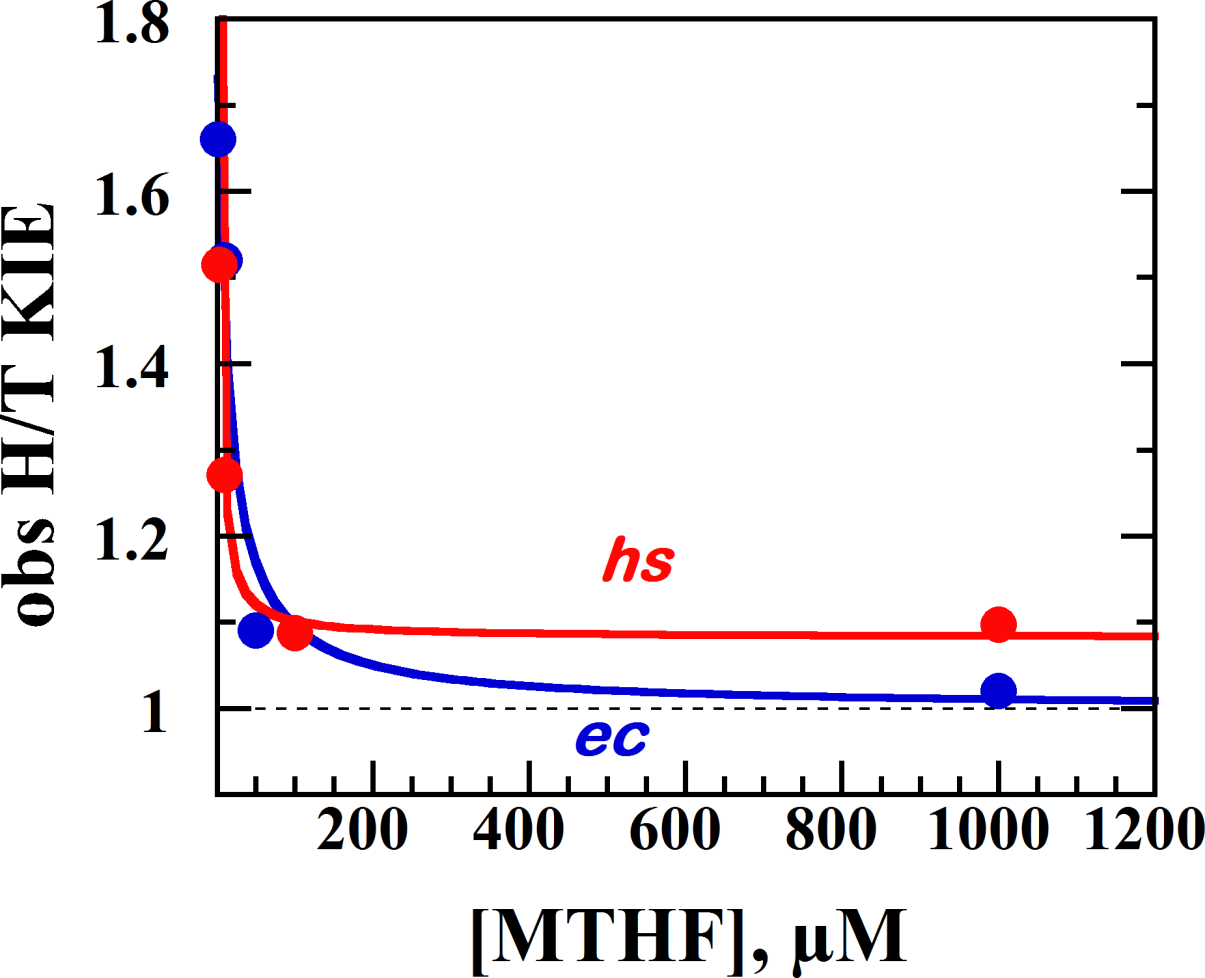
Observed KIEs on the proton abstraction vs the concentration of MTHF for *ec*[19] and *hs*TSase.

However, in the scenario where the binding preference for the ligands is less strict, both substrates can dissociate from the ternary complex, reducing the commitment factor and yielding a non-unity KIE even at high concentration of MTHF. KIEs on the proton abstraction with *hs*TSase at MTHF concentrations ranging from 3 μM to 1000 μM indicate that high MTHF concentrations yielded a non-unity KIE, in contrast to a unity KIE for *ec*TSase (Fig 3). The non-unity KIE (1.10 ± 0.02) suggests that *hs*TSase exhibits a slightly less ordered binding than *ec*TSase. It should be noted that most mutations in *ec*TSase studied cause a disruption in the native ordered-binding mechanism [19, 21].

#### Activation of hydride transfer in *E*. *coli* vs. human TSase

In the cascade of chemical transformations catalyzed by TSase, the hydride transfer from the C6 of THF to the C7 of exocyclic methylene intermediate is particularly notable (step 5, Fig 1). A covalent bond between a carbon and hydrogen (C-H) is very strong and stable. Nevertheless, enzymes are capable of efficiently catalyzing the transfer of a hydride between carbons–reactions that do not occur readily in aqueous solution in the absence of the enzyme. Temperature dependence of intrinsic KIEs has proven to be a useful tool to probe the underlying physical and molecular details of a H-transfer in enzyme-catalyzed reactions [38, 41]. The temperature dependence of intrinsic KIEs reflects how tight and accurate the transition state (TS) of the H-transfer in question is. WT *ec*TSase, like many other native enzymes, exhibits temperature-independent intrinsic KIEs for its catalyzed hydride transfer. According to the activated tunneling model, which has been described in detail in several review articles by us [32, 37, 38, 42] and others [41, 43–49], temperature-independent KIEs represents a very tight, well-organized TS for the H-transfer under study with a narrow distribution of distances between the H-donor and the H-acceptor (DADs) or, in other words, a high-frequency ‘vibration’ representing DAD sampling. On the other hand, temperature-dependent KIEs indicate a loosely-held TS with low-frequency of DAD sampling fluctuations. Most WT enzymes including *ec*TSase evolved to efficiently catalyze the hydride transfer by perfecting the TS structure for a difficult step [38]. However, perturbation of TS by mutating residues associated with the H-transfer reaction coordinate or using non-physiological/native substrates causes a broader distribution of DADs, which is reflected as temperature-dependent KIEs.

**Fig 4:**
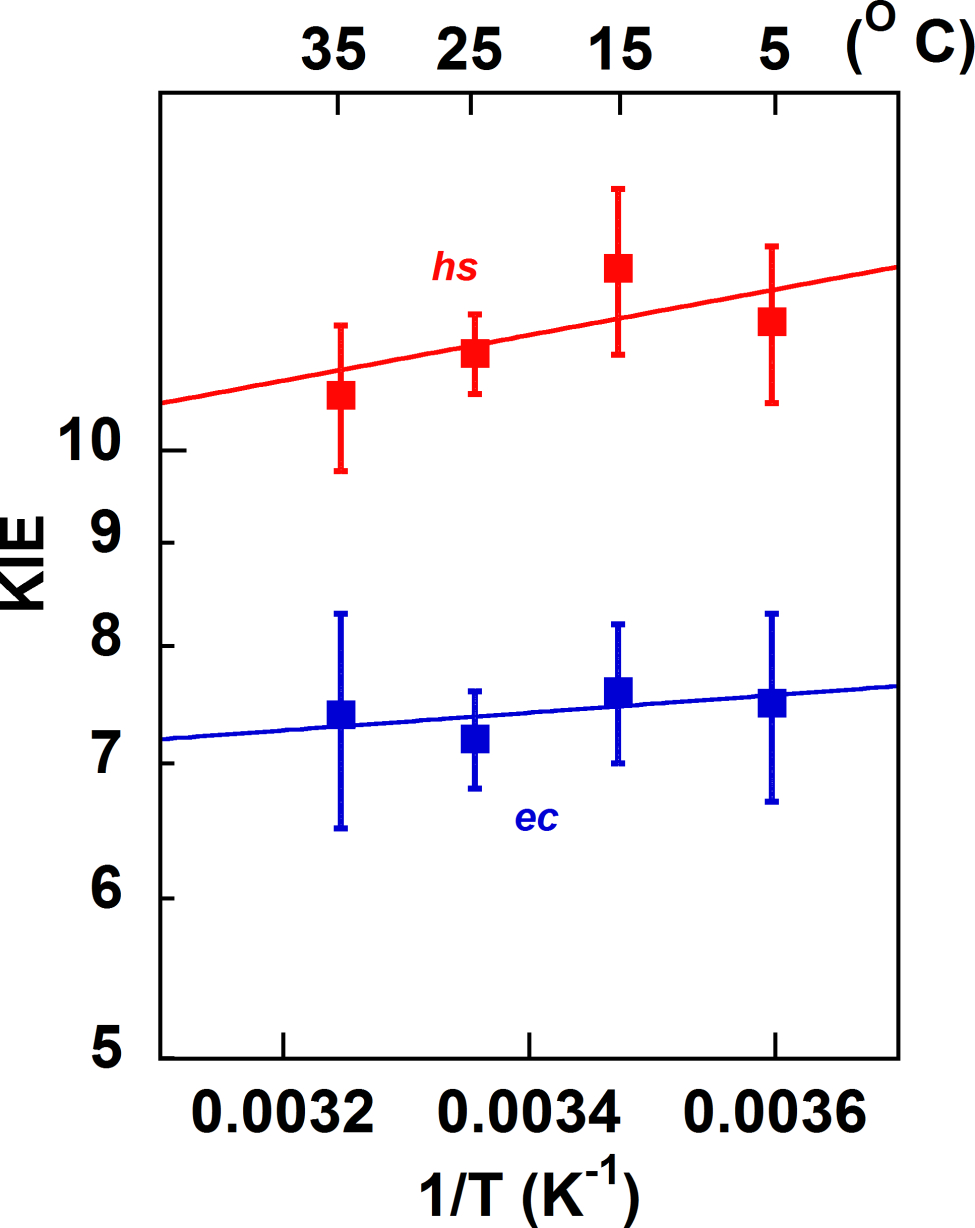
Arrhenius plot of KIE_int_s for hydride transfer. The lines are from regression to Eq 4 for *ec*TSase (blue) [8] and *hs*TSase (red).

To probe the activation of the hydride transfer in *hs*TSase, we measured KIEs at temperatures ranging from 5 to 35 ˚C by a competitive method that reports on the second order rate constant (*k*_cat_/K_*m*_). The observed KIEs are often suppressed by kinetic complexity [39, 50, 51] and therefore are smaller than the actual intrinsic KIEs on bond cleavage *per se*. To assess intrinsic KIEs, a combination of KIEs was measured (see Materials and Methods) and the Northrop method was used [50–52]. Intrinsic KIEs (KIE_int_) for *ec*TSase (blue) and *hs*TSase (red) are shown in Fig 4 as an Arrhenius plot. The Arrhenius equation (Eq 4) was fitted to the intrinsic KIEs, which yielded isotope effects on the activation energy (ΔE_a (T-H)_) and on the pre-exponential factor (A_H_/A_T_) (Table 3) where H and T denotes hydrogen and tritium, respectively.

**Table 3:** Isotope effects on the activation parameters of *ec* and *hs*TSase.

	Hydride Transfer		Proton Abstraction	
	*ec*^*a*^	*hs*	*ec*^*b*^	*hs*
***∆E***_**a,T-H**_ (kcal/mol)	0.2 ± 0.1	0.5 ± 0.1	8.0 ± 0.1	6.0 ± 0.4
***A***_**H**_**/*A***_**T**_	5.6 ± 1.8	2.2 ± 0.2	8.3 (± 1.3) · 10^−6^	3.6 (± 2.3) · 10^−4^

^a^ref [8]

^b^ref [22]

Though Mg^2+^ has a marginal impact on the observed KIEs, it doesn’t appear to have an impact on the intrinsic KIEs of *hs*TSase. Regardless of the presence of Mg^2+^, the intrinsic KIEs on the hydride transfer are temperature independent for *ec*TSase [8, 18], similar to the many other WT systems [53]. However, it seems that *hs*TSase KIEs exhibits a statistically significant temperature dependency of its intrinsic hydride transfer KIEs, with ΔE_a,T-H_ of 0.5 ± 0.1 kcal/mol. Additionally, the magnitude of intrinsic KIEs are larger for *hs*TSase than that of *ec*TSase. According to the activated tunneling models [32, 37, 38, 42], the DAD sampling frequency of hydride transfer in *hs*TSase is lower and the average DAD is longer, both of which suggest a less accurate TS of hydride transfer for the recombinant *hs*TSase. Human enzymes are highly evolved, and therefore transition states for difficult steps are expected to have been optimized for an efficient H-transfer. For instance, human dihydrofolate reductase that catalyzes a hydride transfer from NADPH to dihydrofolate was found to exhibit temperature independent KIEs with smaller intrinsic KIEs than its bacterial counterpart [54]. A possible reason for the less than perfect TS in the recombinant *hs*TSase is that it has been expressed in *E*. *coli* and thus lacks post-translational modifications. It is clear that physiologically, TSase is post-translationally modified in certain cell types and conditions. However, it is not out of the question that the evolutionary pressure is not actually toward increasing activity and hydride transfer efficiency. *E*. *coli* grows quickly and withstands mutations. Human cells divide slowly, and mutations impact their viability. Therefore, if too much thymidylate is produced, there could be an imbalance between dTTP and the other dNTPs. As a result, polymerase error rates could go up, causing mutations. Indeed, “genomic instability” and cell death have been reported as a result of this alteration in relative concentrations of the dNTPs [55, 56]. Therefore, a less active TSase will maintain the proper ratio between dNTPs and minimize mistakes by the DNA polymerases [55, 56]. Thereby, we suspect the temperature-dependent KIE outcome may stem from the fact that the recombinant enzymes used in the current studies may not be physiologically relevant or from the possible evolutionary pressure to avoid overproduction of thymidylate. Investigations of this sort on TSase of organisms that are evolutionarily between *E*. *coli* and human do not appear to have been published. Initiatives are underway to express this enzyme in mammalian cells to assess any posttranslational modifications and their impact.

#### Activation of proton abstraction in *E*. *coli* vs human TSase

**Fig 5:**
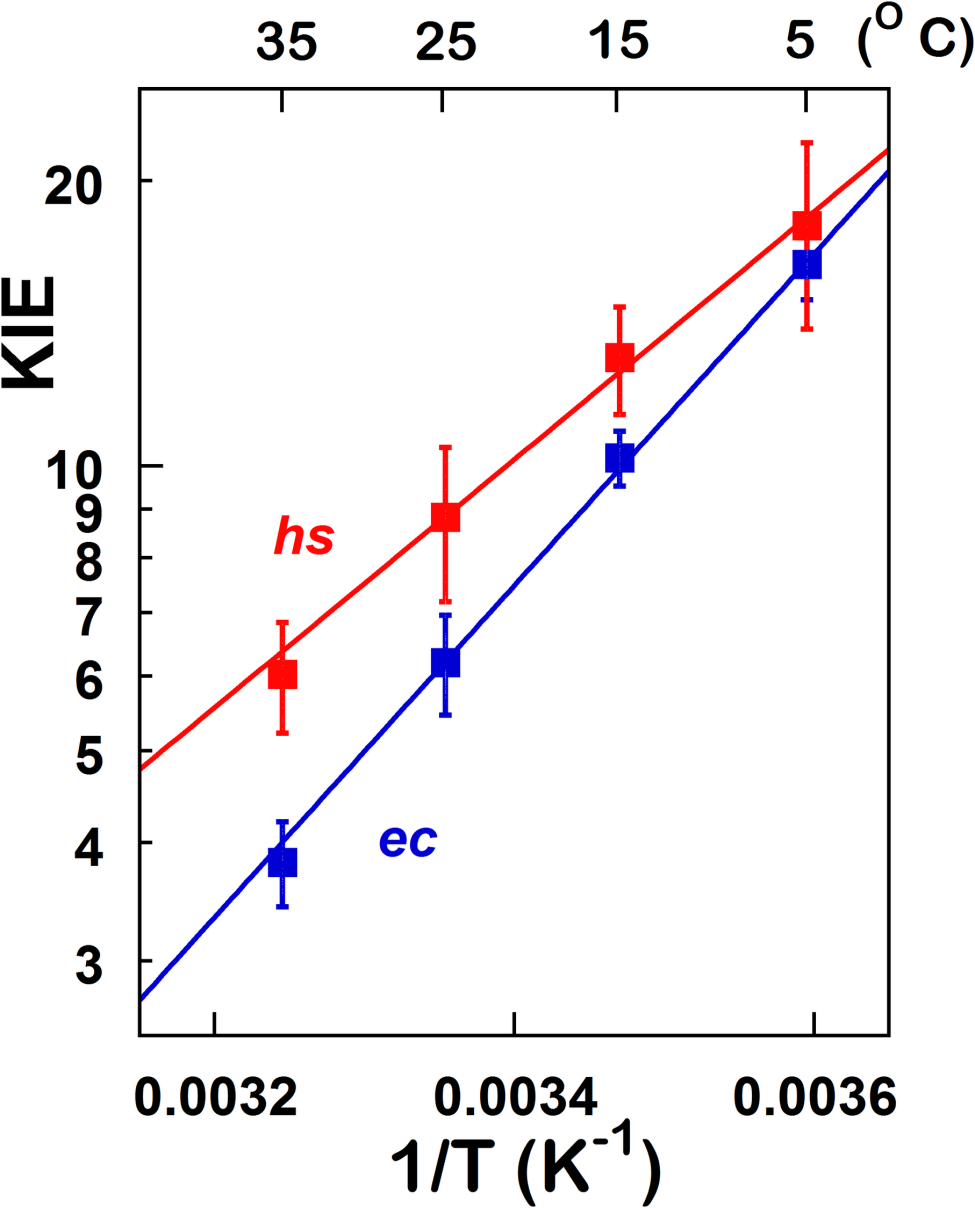
Arrhenius plot of KIE_int_s on the proton abstraction. [*ec*TSase data from [22]].

To probe the activation of the proton abstraction in *hs* TSase, we also examined this step by measuring KIEs and their temperature dependence. The observed KIEs on the proton abstraction depend on the concentration of the MTHF (Fig 3). Because of the large commitments at high concentration of MTHF, only 4 μM MTHF was used. We measured both H/T and D/T observed KIEs and extracted intrinsic KIEs by the Northrop method as reported for the *ec*TSase [22]. As can be seen in Fig 5 and Table 3, in contrast to the hydride transfer, the intrinsic KIEs on the proton abstraction for *ec*TSase are steeply temperature dependent, suggesting a loose transition state for the proton abstraction. Unlike the hydride transfer where a well-defined species is the hydride acceptor, a network of hydrogen bonded residues [29] including water acts as a proton acceptor. As discussed in more detail in ref [22], this can be understood as a step requiring less catalytic enhancement by the enzyme, and thus its TS need not be “perfect.” Indeed, at 1 M cysteine and pH ~8–9 in room temperature aqueous solution, there is exchange of the C5 position as a result of Michael addition by the thiolate [30]. For *hs*TSase, the intrinsic KIEs are less steeply temperature dependent than for *ec*TSase [22], suggesting a less loose transition state for the proton abstraction in the mammalian enzyme.

## Conclusions

TSase is a highly conserved enzyme from bacterial to human sources. A significant portion of the primary sequence, tertiary structure, and chemical mechanisms also seem to have been conserved in most of the organisms encoding the *thyA* gene throughout the evolutionary spectrum. Since bacterial TSases are more accessible and easier to study than their human counterpart, a plethora of kinetic, structural and mechanistic studies have been performed on TSases from *E*. *coli* and *L*. *casei* [4]. Bacterial TSases has thus been used as a model system for drug design. Despite having high-level similarities in structure and functions, TSase appears to possess some species-specific properties. Bacterial and mammalian cells contrast in the intracellular concentration of Mg2+–the former carries a relatively higher level of Mg^2+^ than the latter [57–59]. Previous kinetic studies indicated that Mg^2+^ increases the reaction and hydride transfer rate for *ec*TSase [8], but the current kinetic studies indicate that this is not the case for *hs*TSase. Mg^2+^ also does not seem to have any significant effects on the binding of the substrates for *hs* TSase–to the extent that *K*_*m*_ values can be judged to reflect binding. The rate enhancement by Mg^2+^ could be crucial for bacterial organisms to provide dTMP for multiplication of cells. The differences in sensitivity to Mg^2+^ may be exploited in designing antibiotic drugs with no toxicity. Specifically, substrates with an abrogation of the carboxylate(s) of the para-aminobenzoyl glutamate tail that serve as a handle for Mg^2+^ binding [8] would be anticipated to preferentially impact bacterial TSase rather than human TSase. Namely, a methylene tetrahydrofolate (MTHF) derivative missing one of the carboxylates of the PABA tail will bind in the active site of both human and *E*. *coli* TSase. The human TSase will be unaffected, but the bacterial TSase will be slowed down.

Bacterial and mammalian TSases also exhibit differences in other respects. While *ec*TSase exerts sequential ordered substrate binding control (dUMP followed by MTHF), *hs*TSase seems to have more flexibility in the binding order of substrates. Differences are also observed in the activation of the hydride transfers between these two enzymes. The hydride transfer for *ec*TSase showed temperature independent intrinsic KIEs, while *hs*TSase shows more temperature dependent intrinsic KIEs, suggesting the former possesses an optimized TS while the latter exhibits a looser TS. However, a less accurate TS for a difficult step was not anticipated from a highly evolved enzyme, raising suspicion regarding the physiological relevancy of *hs*TSase when expressed in an *E*. *coli* host system. However, we must not rule out evolutionary pressure driving the enzyme catalytic efficiency and hydride transfer TS efficiency down in order to balance the nucleotide pools and avoid the misincorporation of nucleotides by polymerases. To increase the likelihood of incorporation of natural posttranslational modifications, efforts are underway to express the *hs* TSase in a mammalian cell line.

## Supporting information

S1 Fig Initial velocities for *hs*TSase vs. [MTHF]. The experiment was performed at [dUMP] = 100 μM. See Materials and methods section for additional details.
(TIF)

S2 Fig Initial velocities for *hs*TSase vs. [dUMP]. The experiment was performed at [MTHF] = 100 μM. See Materials and methods section for additional details.
(TIF)

S1 Table Observed KIEs related to Fig 3. (XLSX)

S2 Table Observed and intrinsic KIEs related to Fig 4. (XLSX)

S3 Table Observed and intrinsic KIEs related to Fig 5. (XLSX)

S4 Table Steady-state rate measurements related to S1 and S2 Figs. (XLSX)
