# Supplementary figures and images for: Bacterial versus human thymidylate synthase: Kinetics and functionality
Source: PLoS One. 2018 May 1;13(5):e0196506. doi: 10.1371/journal.pone.0196506 (PMC5929524; doi:10.1371/journal.pone.0196506)

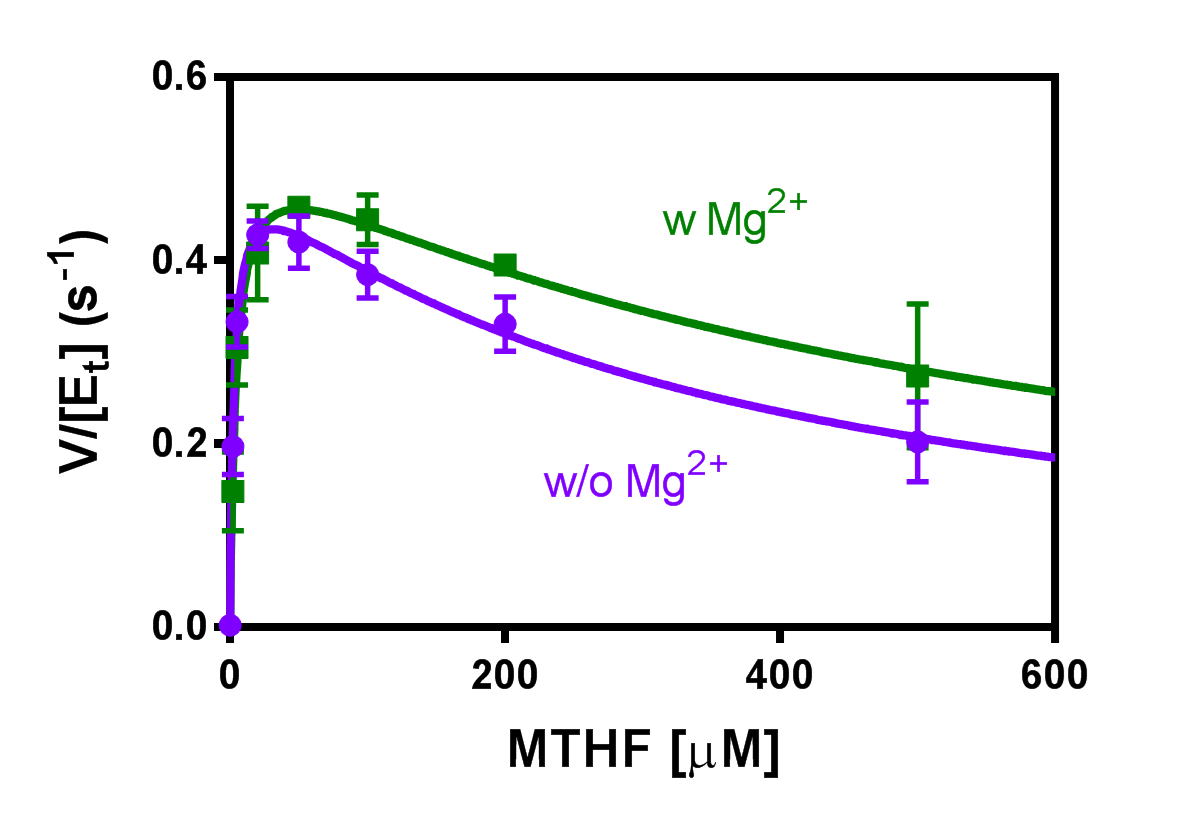

Supplement: S1 Fig — The experiment was performed at [dUMP] = 100 μM. See Materials and methods section for additional details. (TIF) [file pone.0196506.s001.tif]

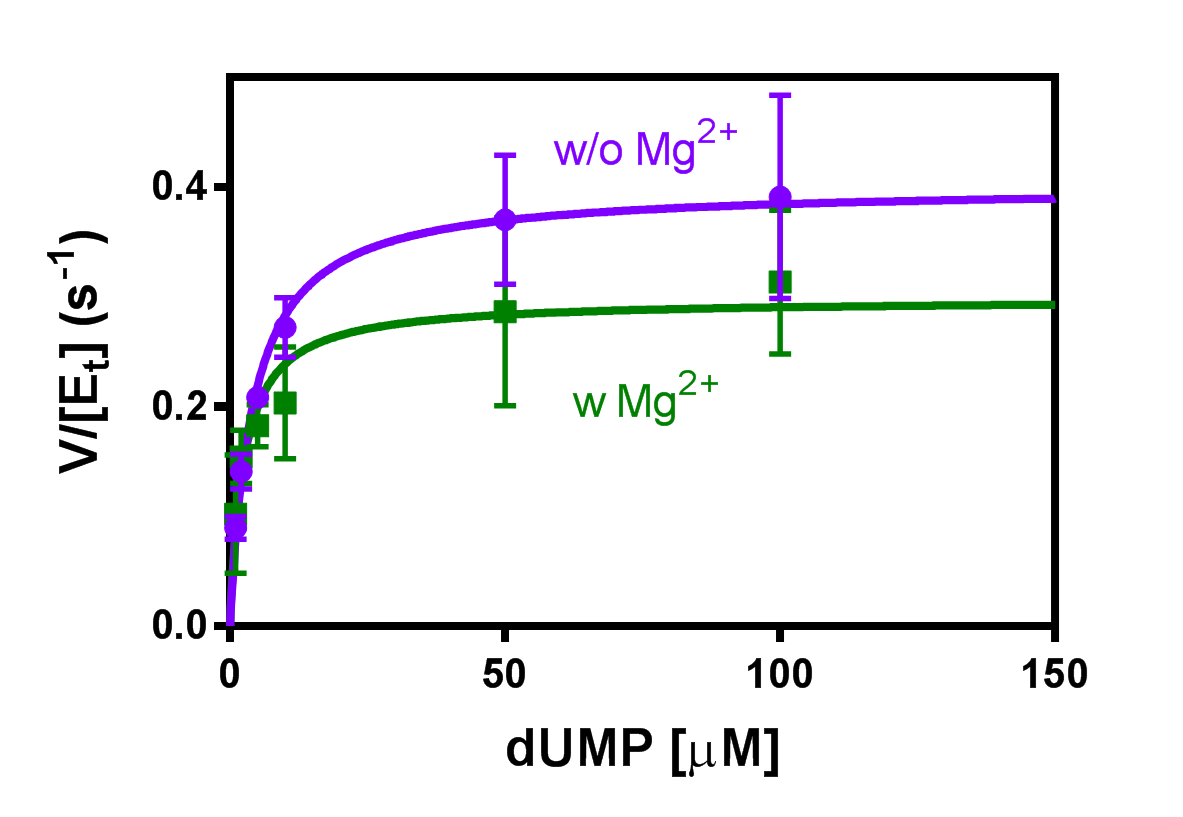

Supplement: S2 Fig — The experiment was performed at [MTHF] = 100 μM. See Materials and methods section for additional details. (TIF) [file pone.0196506.s002.tif]
